# Long-term forecasts of the COVID-19 epidemic: a dangerous idea

**DOI:** 10.1590/0037-8682-0481-2020

**Published:** 2020-08-26

**Authors:** Edson Zangiacomi Martinez, Davi Casale Aragon, Altacílio Aparecido Nunes

**Affiliations:** 1 Faculdade de Medicina de Ribeirão Preto, Universidade de São Paulo, Ribeirão Preto, SP, Brasil.

**Keywords:** COVID-19, Coronavirus disease, Forecasting, Statistical models, Epidemiology

## Abstract

**INTRODUCTION:**

Mathematical models have been used to obtain long-term forecasts of the COVID-19 epidemic.

**METHODS:**

The daily COVID-19 case count in two Brazilian states was used to show the potential limitations of long-term forecasting through the application of a mathematical model to the data.

**RESULTS:**

The predicted number of cases at the end of the epidemic and at the moment that the peak occurs, is highly dependent on the length of the time series used in the predictive model.

**CONCLUSIONS:**

Predictions obtained during the course of the COVID-19 pandemic need to be viewed with caution.

In December 2019, in Wuhan, China, a new beta coronavirus was discovered. In January 2020, the World Health Organization declared this outbreak to be a global health emergency and named the correspondent disease as 2019 coronavirus disease (COVID-19). Since then, global efforts are being made to find solutions for the management of COVID-19. Among other important contributions, mathematical and statistical models are being used to forecast the short and long term course of the COVID-19 epidemic in a given population; these results are useful for estimating medical capacity requirements and to keep the public and decision-makers informed. However, it is well-known that these forecasts are very difficult, as they hinge critically on the change of epidemiological parameters in response to interventions^1^. Forecast models are based on the premise that, “the most reliable way to predict the future is understand the present” and, for this reason, these models do not say what will actually happen in the future, but say what can happen if the conditions observed in the present do not change. Based on this idea, in mid-March 2020, Prof. Neil Ferguson and his colleagues at Imperial College’s MRC Centre for Global Infectious Disease Analysis presented the results from a mathematical model that indicated that the United Kingdom’s health service would soon be overwhelmed with severe cases of COVID-19 and more than 500,000 deaths, if the government did not take action^2^ ^,^ ^3^. This model also suggested that, in the absence of action, 2.2 million people would die from the disease in the United States^3^. These predictions were based on some assumptions regarding the natural history and clinical management of the COVID-19 epidemic, including incubation period, infectiousness before symptom onset, mean generation time, and the basic reproduction number^2^. However, during a pandemic it is very difficult to get reliable data, especially in cases where knowledge about the disease and the biopathogenic characteristics of its etiological agent, is limited. The Imperial College model was criticized for not utilizing actual data, but Ferguson defended the results by arguing that “models are not crystal balls”, but tools to provide simplified representations of reality^3^ ^,^ ^4^.

During the course of the COVID-19 pandemic, a number of authors used simpler models than the one proposed by the Imperial College to attempt long-term forecasts of the number of cases^5^ ^,^ ^6^. However, many of these models are based only on mathematical premises, while there are many unquantifiable factors like changes in public health policies, dynamics of the disease, and the biological and sociodemographic characteristics of the population, that can substantially affect long-term forecasts. A common strategy is to model the cumulative number of cases of COVID-19 on an S-shape (Sigmoid) growth curve and thus graphically observe the behavior of the curve in the following days. These curves are usually based on well-known cumulative distribution functions, such as those corresponding to the Gompertz, logistic, log-normal and Gumbel distributions^7^. As a special case, the Richards growth curve assumes that the cumulative number of cases of the disease at time t is indicated by the expression:

$$C\left(t\right)=K{\left\{1+\exp\left[-ra\left(t-b-\frac{\ln a}{ra}\right)\right]\right\}}^{-1/a}$$

The structure of this equation is amenable to infectious disease modeling, since its parameters have direct interpretations. K is the cumulative number of cases at the end of the epidemic, r is the per capita growth rate of the cumulative number of cases, a is the exponent of deviation of the cumulative case curve, and b is the turning point, or the moment at which the peak occurs^8^. To illustrate our point, we take the official number of daily reported cases in the Brazilian states of São Paulo (SP) and Ceará (CE) from the date of notification of the first case in each state, up to July 8, 2020. These data were obtained from the Brazilian Health Ministry^9^. The first cases of COVID-19 in SP and CE were reported on February 25 and March 16, respectively. Panels (a) and (b) of Figure 1 compare the actual cumulative number of daily reported cases with the curves fitted by a Richards model considering normal errors. For applying this model, we used the nls function (nonlinear least squares) of the R language (version 3.6.2). For both states, we observe a good fit of the model to the data, given that the estimated growth curves are close to the actual values.

**FIGURE 1:**
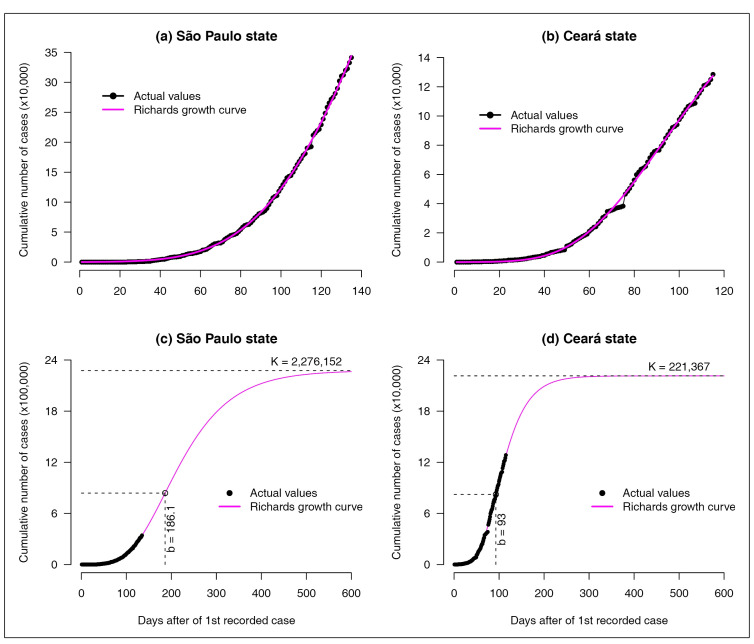
Panels **(a)** and **(b)** show plots of the cumulative number of daily COVID-19 cases from the date on which the first case was notified in the São Paulo and Ceará states, respectively, up to July 8, 2020. The graphs compare the actual values and the correspondent values predicted by a Richards model. Panels **(c)** and **(d)** show long-term forecasts for the São Paulo and Ceará states, respectively, based on the Richards growth model. *K* denotes the cumulative number of cases at the end of the epidemic and b denotes the date that the peak occurs (the inflection point of the curve).

However, the fact that a model is capable of providing a curve very close to the actual data, does not mean that it is useful for making predictions. Considering the model based on the Richards curve, it is estimated that in SP there will be K = 2,276,152 cases of COVID-19 by the end of the epidemic, and the peak of cases will occur on day b = 186.1 (tentatively, August 28, 2020). In addition, it is estimated that in CE there will be K = 221,367 cases of the disease by the end of the epidemic, and that the peak of cases occurred on b = 93 (June 16, 2020). Presented in the lower panels of Figure 1 are the projections of the growth curves during a period of 600 days. Although the actual values and those obtained from the fit of the Richards model are quite close (as shown in Figure 1), there is no guarantee that the epidemic curve will continue to grow according to this mathematical model after the period used to adjust the curve. Therefore, these estimates for K and b obtained from the Richards model, although correct from a mathematical perspective, are highly unrealistic.

In order to demonstrate this statement, we fit the Richard model to data from SP and CE, considering the daily reported cases from the date of notification of the first case in each state, up to three different dates: May 28, June 10 and June 29, 2020. Figure 2 compares the projections of the obtained growth curves over a period of 600 days for SP and 400 days for CE. We can observe in panels (a) and (b) of figure 2 that the estimates of the cumulative number of cases at the end of the epidemic K, and at the moment of occurrence of the peak b, vary widely according to the period considered. In both states, though more pronounced in CE, a decrease in the daily COVID-19 reports was observed close to May 28, followed by a sudden increase in the records. This was probably due to delays in diagnosis or in notification, but was enough to produce a false impression that the peak would occur soon, as shown in Figure 2. In CE, the daily COVID-19 reports increased significantly close to June 10, but a deceleration in diagnosis (or notifications) was observed in the following days, which may be a consequence of social isolation measures, and/or reduced testing. Thus, Figure 2 shows that the forecasts on June 10 in CE produce a more pessimistic scenario for the disease than the forecasts on a posterior date (say, June 29).

**FIGURE 2:**
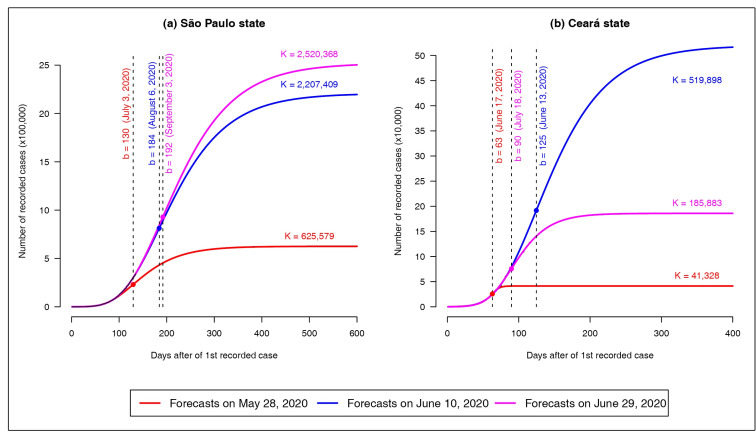
Long-term forecasts for **(a)** São Paulo and **(b)** Ceará based on the Richards growth model, in three different scenarios: forecasts on May 28, June 10 and June 29, 2020.

Figure 3 shows estimates of parameters K and b obtained from the fit of Richards models to the daily COVID-19 reports in SP and CE, considering a time series beginning on the date of notification of the first case in each state and ending on different dates, in a range from April 14 to July 8, 2020. Considering the high variation of the estimates shown in these graphs, these findings reinforce the conclusion that, during an epidemic (at least mathematically), the prediction of the number of cases at the end of the epidemic and at the moment of occurrence of the peak is highly dependent on the number of days used in the predictive model. That is, all other important variables, such as the natural history of the disease, population biological and sociodemographic characteristics, as well as public policies for mitigating the epidemic, are completely unforeseen by the model.

**FIGURE 3:**
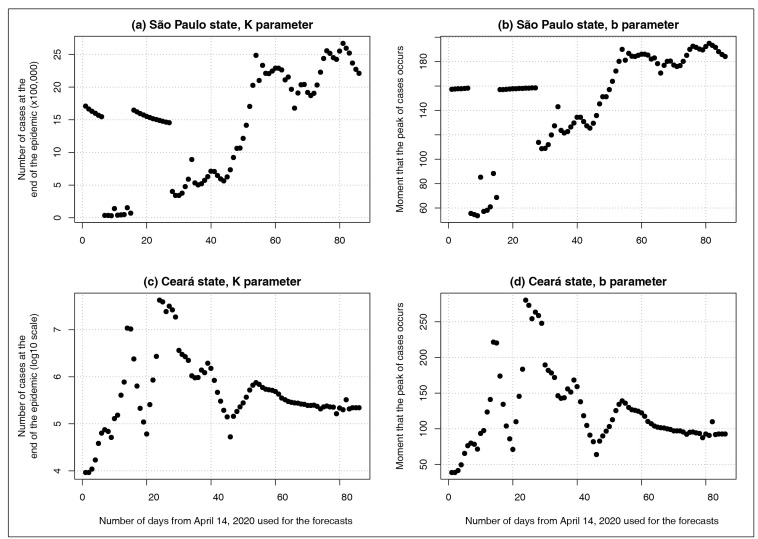
Estimates of the parameters K and b, obtained from the fit of Richards models to the number of daily COVID-19 cases in São Paulo [panels **(a)** and **(b)**] and Ceará [panels **(c)** and **(d)**]. It was considered as a time series beginning on the date of the first case notified in each state and ending in a range from April 14 to July 8, 2020. Estimates of K in panel **(c)** are described in logarithmic scale due to a highly skewed distribution.

Therefore, models based on S-Shape curves are more appropriate to describe the dynamics of an epidemic after its abatement. If they are used at the beginning of the epidemic, just to obtain a smoothened curve of the cumulative number of cases, care must be taken with the interpretation of their parameters. Short-term forecasts can be obtained from the immediate trajectories of the curves obtained from these models, which are likely to be more accurate than long-term forecasts, but are also sensitive to the high volatility observed at the end of the time series of reported cases^10^. These variations occur due to extrinsic factors, such as the availability of tests for essential screening, the natural history of the disease and changes in mitigation measures. Using an S-Shape curve model, Faranda et al.^11^ demonstrated the high sensitivity of the estimates to the last point of COVID-19 datasets. These authors provide a simulation study, replacing the last data point of the epidemic curves in the UK, France and Italy with a random number drawn from a uniform distribution, showing that the trajectory of the curves obtained under this process have a very high variability. Faranda et al.^11^ also showed that long-term forecasts and predictions based on more sophisticated models, such as the Susceptible-Exposed-Infected-Recovered (SEIR) compartmental model, are also extremely sensitive to biases in data collection and crucially depend on the last available data point.

Thus, during its course, the future of an epidemic in a real population is unpredictable due its natural dependence on a broad number of variables. The use of more sophisticated mathematical tools require a minimal number of premises to obtain less biased estimates. These premises include the necessity of accurate information on the number of susceptible, infected, exposed and recovered people, which is extremely difficult to obtain in any country^12^. Among the sources of uncertainty, we have underreporting and delays in reporting cases; inaccuracies in the estimates of the percentage of people that comply with measures of social distancing and wearing masks; unavailability of tests and lack of accuracy of test methods; limited knowledge about herd immunity and the mechanism that enables oligosymptomatic or asymptomatic individuals to transmit the disease; the incubation period of the virus; and other factors^13^ ^,^ ^14^. Declaring all the mathematical assumptions of a model is essential but is not sufficient for an adequate interpretation of the results. An extensive discussion of these premises is essential in any scientific work aimed at forecasting cases of COVID-19.

At the same time, it is necessary to develop scientific literacy for all citizens, since the constant appearance of epidemic curves and predictions in newspapers and electronic media has made these tools popular with the general population. In a quote attributed to the American business tycoon Warren Edward Buffett, one of the most successful investors worldwide, it is stated that "forecasts may tell you a great deal about the forecaster; they tell you nothing about the future"^15^. If in the business world, predictions need to be viewed with caution as they essentially express an investor’s point of view, in an epidemic the prediction of peak cases, the possible flattening of the epidemic curve or the date of the end of the epidemic can also just be someone's guess, and may not necessarily be a scientific prediction of the future, obtained from mathematical modeling. 

In conclusion, remembering the premise that “all models are wrong, but some are useful”, a quote attributed to the British statistician George Box, adequate COVID-19 epidemic forecasts require a deep understanding of mathematical, statistical and epidemiological methods, and their assumptions and premises must be adequately verified and validated by experts.
